# The effect of lockdowns and distant learning on the health-related behaviours of school students in the United Arab Emirates

**DOI:** 10.1186/s12875-022-01856-y

**Published:** 2022-09-27

**Authors:** Najla Hussain Sajwani, Ahmed Qawas, Nouf Al Ali, Fatma Hussain Sajwani, Asma Hamed Alrustamani, Shamma Al Maamari, Shereena K. Al Mazrouei, Budoor Al Shehhi, Hussain Al Rand, Asma Mahmoud Fikri

**Affiliations:** 1 Primary Healthcare Department, Emirates Health Services, Dubai, United Arab Emirates; 2 Statistics and Research Centre, Ministry of Health and Prevention, Dubai, United Arab Emirates; 3 Public Health Sector, Ministry of Health and Prevention, Dubai, United Arab Emirates; 4 Laboratory Department, Emirates Health Services, Dubai, United Arab Emirates; 5 Education Policy Department, Ministry of Education, Dubai, United Arab Emirates; 6 Abu Dhabi Public Health Centre, Abu Dhabi, United Arab Emirates; 7 National Centre for Health Research, Ministry of Health and Prevention, Dubai, United Arab Emirates; 8 Faculty of Medicine, University of Sharjah, Sharjah, United Arab Emirates

**Keywords:** COVID-19, School Students, Health related habits, Children behaviour, Physical activity, Screen time

## Abstract

**Background:**

The COVID-19 pandemic began to affect the world in early 2020. As a preventive measure, schools in the UAE adopted remote learning. This study aimed to assess the effects of the lockdown and remote learning on the health-related behaviours of school students in the UAE.

**Methods:**

A cross-sectional study using an online survey comprising 33 questions related to physical activity, eating, sleeping and screen time was answered by the students’ parents. Chi-square tests, paired Student’s t tests and frequency tables were used for analysis.

**Results:**

A total of 27,754 responses were received: 46.3% of the parents indicated a significant decrease in physical activity; 44.6% indicated an increase in unhealthy snack consumption; and 51.9% indicated decreased food ordering from restaurants. The percentage of students who slept more than 9 hours and those who slept less than 6 hours increased. Screen time increased significantly for both educational and entertainment purposes (*P* value < 0.0001).

**Conclusion:**

The study showed changes in the lifestyle and health-related behaviours of school students as indicated by their parents. Risk factors such as a lack of physical activity, increased food consumption, sleeping and screen time were directly affected. Therefore, it is important to further investigate these changes and their effects to help design targeted health education programs and promotion campaigns.

## Background

COVID-19 began to affect the world’s population in early 2020. It was declared a global pandemic by the WHO in March 2020 [1]. Many countries around the world implemented different measures with the aim of minimizing the spread of the infection. These mitigation measures included wearing face masks and maintaining a physical distance of 6 ft. In March 2020, the United Arab Emirates (UAE) made the decision for all schools to shift to remote (online) learning to limit crowding in schools to protect children and the community, hence decreasing the load on the health care sector.

Home schooling, lockdowns, and social distancing are important measures in containing the ongoing pandemic [2]. However, published reports have shown that these measures significantly affect people in many different ways [3–8]. A side effect of the pandemic and its control measures is the impact that home schooling and distant learning have had on school children’s health-related behaviours across the world [3–8]. These changes in behaviours can be risk factors for many health problems, including obesity. Children with obesity are prone to a number of diseases, such as cardiovascular diseases, hypertension, diabetes, metabolic syndrome, sleep apnoea, gastrointestinal problems and certain cancers [9].

The WHO Global Observatory data suggest that the Eastern Mediterranean region has the highest prevalence of physical inactivity in adolescents (87%) among WHO regions [10]. In the UAE, according to the 2018 Report Card on Physical Activity, only 16% of UAE children achieve the daily physical activity recommendation of more than or equal to 60 minutes of moderate to vigorous physical activity (MVPA) across age groups and sexes [11]. According to a study conducted in Abu Dhabi in 2019, the majority of boys and girls are not meeting the recommendation of 30 min/day of MVPA during the school day, with girls accumulating less activity and more sedentary minutes than boys [12]. This is expected to worsen with lockdowns and home schooling.

Obesity among children was recognized as a public health problem in the UAE before the pandemic. Studies and reports have shown that 38–41% of school children aged 13–17 years are overweight, while 17–24% are obese [13, 14].

The UAE was one of the first countries to adopt recommended public health measures in its pandemic response. Part of the health care sector’s responsibility was to conduct national surveillance and online surveys to assess the needs of the public considering the ongoing pandemic and to identify high-risk groups and changes in social and health-related behaviours. This study was conducted in the UAE by the Health Centers and Clinics sector at the Ministry of Health and Prevention (MOHAP) in collaboration with the Ministry of Education (MoE), Abu Dhabi Department of Education and Knowledge (ADEK) and Abu Dhabi Public Health Center (ADPHC). The study aimed to assess the effect of the lockdown and remote learning on the health-related behaviours of school students in the UAE, including physical activity levels, eating habits, sleeping hours and the time students spend in front of screens for educational and noneducational purposes.

It is essential to understand the impact of the pandemic and preventive measures on the health of school students. Understanding the effects will help determine necessary corrective measures and improve future pandemic responses [2].

## Methods

### Study design

A cross-sectional quantitative online survey comprising 33 questions was sent to the students’ parents or guardians for completion during the period from August to December 2020. Survey questions included the parents’ and children’s demographic variables; duration and type of physical activity; eating behaviours including unhealthy snack and sweetened beverage consumption and food ordering from restaurants; sleeping hours; and screen time. The questions addressed these health-related behaviours at two time points: before the pandemic and during the pandemic (in the 4 months of completing the questionnaire; August to December 2020).

### Ethical considerations

The study was reviewed and conducted following an approval exemption granted by the MOHAP Research Ethics Committee.

### Study population and eligibility

An electronic survey link, including forms of the survey in both Arabic and English, was distributed by the MoE and ADEK to the registered parents of all school students, from grade 1 to grade 12, in Abu Dhabi and the Northern Emirates.

In our study, there were more responses from parents of children in private schools than government schools because the private schools sent the survey repeatedly and reminded the parents about the importance of their response, while the government schools only sent the survey to the parents once and did not send any reminders.

### Data collection and statistical analysis

This cross-sectional survey was distributed during the period between August and December 2020. A total of 27,754 responses were received. From the original 27,754 participants who completed the survey, 27,729 participants provided data for all the relevant study variables, and 25 participants were excluded due to missing values.

Data were processed as follows: the first part of the data analysis included correlating the demographic variables with physical activity status using chi-square tests (independent demographic variables were studied for any significant relationship with or effects on the following dependent variables: physical activity level, screen time, healthy eating behaviours and sleeping hours).

Paired samples Student’s t test was used to determine the *P* value. This approach was employed to determine any significant differences between the demographic groups and before and after the COVID-19 pandemic. *P* values are indicated with asterisks, and the number of asterisks indicates the level of significance shown as follows: *P < 0.05; **P < 0.01; ***P < 0.001.

In the second part of the analysis, frequency tables were created for all dependent variables to analyse the overall outlook on the change within these variables before and after the start of the pandemic.

Data analysis and statistics were performed using Microsoft Excel and SPSS® Statistics (IBM) version 26.0.

## Results

A total of 27,754 responses were received. The data were analysed, and statistical analysis was performed as described in the Methods section. From the original 27,754 participants who completed the survey, 27,729 provided data for all the relevant study variables and were included in the final analysis (Table 1). Both parents were represented almost equally in the sample (55.7% men and 44.3% women), with the majority being in the age group of 31–40 years (49.3%). UAE citizens represented 27% of the sample, while expatriates accounted for 73%. Regarding the children’s demographic variables, 17.8% of the children were kindergarten students (the lowest percentage), followed by high school students (19.5%) and secondary school students (27.2%), and elementary students represented the highest percentage (38.5%). A total of 16.6% of the students were attending government schools. In private schools, 34.1% of the students were studying the government curriculum, and 49.3% were studying foreign curricula (Table 1).

**Table 1:** Survey participants’ sex, age, and their children’s school demographic variables expressed in numbers and percentages of the total responses (*n* = 27,729)

Characteristic	Category	Frequency	Percent
**Sex**	Female	15,446	55.7%
	Male	12,283	44.3%
**Age**	Below 20 years old	1126	4.1%
	20–30 years old	1989	7.2%
	31–40 years old	13,694	49.3%
	41–50 years old	9084	32.7%
	51 years old or above	1836	6.7%
**Nationality**	UAE citizen	7460	27.0%
	Expatriate	20,268	73.0%
**Child’s Grade**	Kindergarten (4–6 years old)	4108	14.8%
	Elementary School, Grades 1–4 (6–9 years old)	10,673	38.5%
	Secondary School, Grades 5–8 (10–13 years old)	7548	27.2%
	High School, Grades 9–12 (14–17 years old)	5400	19.5%
**Child’s School Type**	Government Education	4588	16.6%
	Private Education implementing a Foreign Education System	13,688	49.3%
	Private Education implementing the Federal Government system	9453	34.1%

**Fig. 1:**
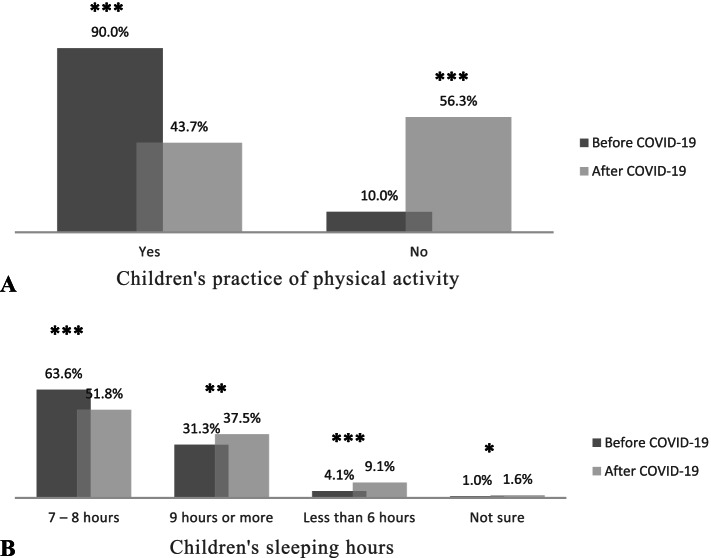
**A** Parents’ responses to the survey question of whether their children practised physical activities before and after the start of the COVID-19 pandemic; **B** Children’s sleep duration before and after the start of the COVID-19 pandemic and the introduction of public health measures and distant learning as indicated by their parents. Data were compared using paired Student’s t test, and differences in mean values are highlighted with asterisks as follows: *P < 0.05; **P < 0.01; ***P < 0.001

In this survey, 90% of the parents indicated that their children practised physical activities before the pandemic, while 43.7% indicated that their children were physically active after the start of the COVID-19 pandemic and the implementation of preventive public health measures. The figure shows a significant decrease of 46.3% in the physical activity of the students after the start of the pandemic compared to before the pandemic (Fig. 1A). Furthermore, it was important to determine any changes in the children’s sleeping patterns. Based on the parents’ responses to the survey, the percentage of students from all age groups who slept 7–8 hours per day decreased significantly after the start of the COVID-19 pandemic, while the percentage of students who slept 9 or more hours and less than 6 hours increased after the start of the COVID-19 pandemic and the subsequent health measures (as shown in Fig. 1B).

**Fig. 2:**
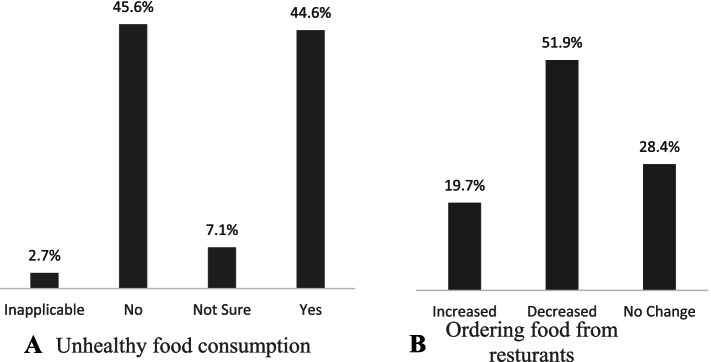
**A** Parents’ responses to the survey question regarding whether they thought their children’s consumption of unhealthy snacks increased after the start of the COVID-19 pandemic and the application of preventive measures compared to before the pandemic; **B** Parents’ responses to the survey question regarding whether they thought the frequency of ordering food from restaurants increased after the start of the COVID-19 pandemic and the application of preventive measures compared to before the pandemic

When asking the parents whether they thought their child’s consumption of unhealthy snacks (foods low in nutritional value and high in fat, sugar and calories) increased after the start of the COVID-19 pandemic and the application of preventive measures compared to before the pandemic, almost half (44.6%) indicated that the consumption of unhealthy snacks increased after the pandemic and lockdown, while the other half (45.6%) reported the opposite (a decrease in consumption), as shown in Fig. 2(A). Additionally, approximately half of the parents (51.9%) indicated that the frequency of ordering food from restaurants decreased after the start of the pandemic, while 19.7% reported that it increased. Furthermore, 28.4% of the parents reported that the frequency stayed the same (Fig. 2B).

**Fig. 3:**
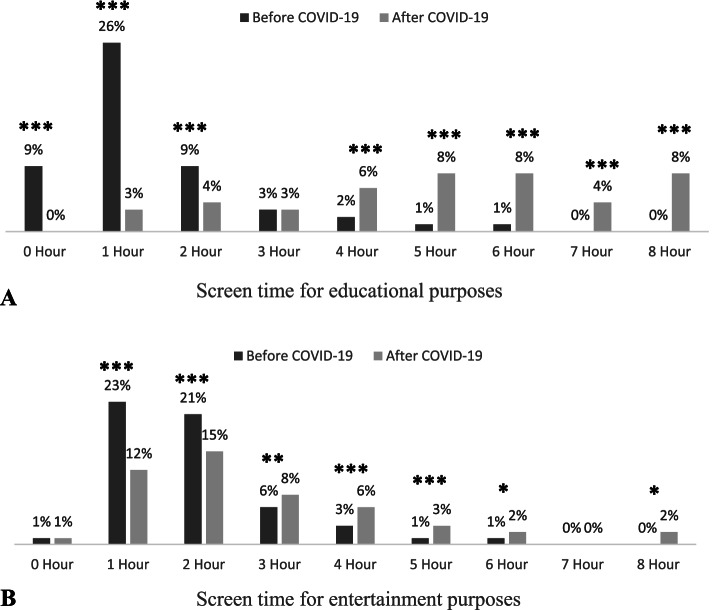
**A** Parents’ answers regarding the amount of time their child spent in front of digital screens for educational purposes before and after the start of the COVID-19 pandemic and the introduction of preventive public health measures and distant learning; **B** Parents’ answers regarding the amount of time their child spent in front of digital screens for entertainment purposes before and after the start of the COVID-19 pandemic and the introduction of preventive public health measures and distant learning. Data were compared using paired Student’s t test, and differences in mean values are highlighted with asterisks as follows: *P < 0.05; **P < 0.01; ***P < 0.001

Finally, this study shows that screen time for educational purposes increased significantly after the start of the COVID-19 pandemic (8% increase in students spending 8 hours in front of a screen for online learning) (Fig. 3A). There was a more than threefold increase in the percentage of students spending 4 hours or more in front of a screen (Fig. 3A). A statistically significant difference before and after the start of the pandemic was observed for hours spent in front of a screen for educational purposes (Mean = 3.52, SD = 2.71; *P* value < 0.0001). On the other hand, the amount of time spent in front of a screen for entertainment purposes decreased for those who spent 1–2 hours in front of a screen prior to the pandemic (Fig. 3B). Additionally, the study found a more than twofold increase in the amount of screen time for children who spent 2 or more hours in front of a screen after the start of the pandemic compared to before the pandemic (Fig. 3B). There was a statistically significant difference observed before and after the start of the COVID-19 pandemic for time spent in front of a screen for entertainment purposes (Mean = 0.95, SD = 2.05; *P* value < 0.0001). There was a noted increase in the number of hours spent in front of a screen for both purposes observed after the start of the pandemic compared to before the pandemic (Fig. 3).

## Discussion

The COVID-19 pandemic has affected many aspects of life, and school students’ lifestyle and health-related behaviours are not an exception. This study aimed to assess the effect of remote learning and quarantine that was adapted by the UAE as a measure to limit the spread of COVID-19 in school children. Data from this study can help in better understanding the effects of the COVID-19 pandemic and guide the implementation of prevention measures that minimize unhealthy lifestyles during the pandemic and in future similar events.

Obesity and one of its main risk factors, a lack of physical activity, was reported as a health concern before the COVID-19 pandemic. A study conducted in the UAE with 44,942 school students showed that the prevalence of obesity and extreme obesity increased linearly each year [13]. According to the indicators of the UAE national agenda (UAE Vision 2021), the prevalence of obesity among children was 17.35% in 2020 [15]. The Global School-based Student Health Survey - 2016 reported that the percentage of obesity in students aged 13–17 years was 16.6% [16]. The WHO published a report in 2014 on promoting physical activity in the EMRO region and identified four major risk behaviours for death due to noncommunicable disease. Two of the risk factors identified were physical inactivity and an unhealthy diet [17]. The current study showed that the implemented preventive measures for COVID-19 also affected these two factors, adding to the risk for the already defined health concern. Parents indicated a decrease in physical activity in 46.3% of the students, while an increase in unhealthy snack consumption was reported by 44.6% of the parents. This is consistent with the decrease in physical activity reported in the general population in the UAE [4, 5]. Working from home was reported to be associated with decreased physical activity as an indirect effect of the COVID-19 lockdown and remote working [4–6].

Similar findings on the effect of virtual learning during the COVID-19 pandemic were reported in other countries in the region. A study from the Kingdom of Saudi Arabia showed a significant increase in the BMI of school students aged 6–18 years after the start of the COVID-19 pandemic compared to before the pandemic [18]. The same study showed increased physical inactivity during the week as well as the weekends. A study from Jordan that compared body weight, physical activity and eating habits in children aged 6–17 years before and after the start of the COVID-19 pandemic showed significant increases in weight and BMI after the lockdown. Physical inactivity was similarly affected. Food consumption increased during the lockdown. This could have contributed to the reported increase in BMI [19].

Other studies have raised the concern that the onset of COVID-19 has aggravated the childhood obesity epidemic, specifically children’s exposure to an “obesogenic” environment that is conducive to physical inactivity and sedentary behaviour, which were found to have a positive association with childhood obesity [20, 21].

The current study showed screen time to be increased significantly for both educational and entertainment purposes (*P* value < 0.0001) during the COVID-19 pandemic, probably due to online learning and staying at home for longer period of time. This was reported by other studies in the general population as a result of the pandemic and consequent lockdowns [3–8]. A similar finding was reported in Jordan, where the time spent watching TV for more than 3 hours increased. Spending more time in front of screens during the day might have replaced the time spent performing physical activity before the COVID-19 pandemic [19].

In our study, ordering ready-made food from restaurants decreased during the COVID-19 lockdown, as reported by 51.9% of the parents. This finding agrees with the findings from Cheikh Ismail et al. [4]; the study showed a decrease in ordering food from restaurants after the start of the pandemic (4.1%) compared to before the pandemic (23.1%) [4]. This might be attributed to the curfew and restrictions of transportation early in the pandemic. Additionally, before the mode of transmission was clarified, people were afraid of being infected by contaminated food during the early phase of the pandemic. Spending a long time at home gave people more time to spend cooking food at home.

In this study, the percentage of school students who slept 9 hours or more increased by 6.2% after the start of the COVID-19 pandemic. Many studies have reported the effect of the COVID-19 pandemic on sleeping patterns in the general population [3–8]. A study reported that sleeping for more than 9 hours per day was significantly increased in the Middle East/African populations during the pandemic [4]. Another study conducted on the UAE’s general population reported that 25.3% of people slept more hours during the pandemic while 20.8% reported a decrease in sleeping hours [6]. According to the American Academy of Sleep Medicine, the recommended number of sleeping hours among school students is between 9 and 12 hours [22]. The observed change in sleeping habits can be a result of limited physical activity, longer hours spent in front of screens, and psychological and mental health issues such as anxiety and depression, which could occur as a result of the pandemic.

This cross-sectional online survey studied different health-related behaviours, such as physical activity level, food consumption (including unhealthy snacks, beverages and ordering from restaurants), sleeping hours and screen time. These factors contribute to many health-related problems, including obesity, sleep disorders, eyesight problems, learning problems, and cognitive and physical development dysfunctions [3–8].

The importance of this study lies in the fact that it was conducted in a region where similar studies are limited. The study population was school students (aged 6–18 years) who accounted for a significant proportion of the population (12.26% in the UAE in the 2019–2020 academic year, which added to the value of the study (Statistics and Research Center, Ministry of Health and Prevention: School Statisitics, Unpublished Report).

Multiple studies have been published showing the effect of the COVID-19 pandemic on lifestyle, behavioural and eating habits in the general population [4–7, 23]. To the best of the authors’ knowledge, although some studies have been published about the effects of the pandemic on school students around the world [7, 8, 23], few have specifically addressed school students in this part of the Eastern Mediterranean region.

### Limitations of this study

The current survey was completed by the parents or guardians of school students. This could contribute to an increased risk of personal bias. In the future, this can be overcome by having two versions of the survey, one directed towards parents and another towards students. Additionally, parents might have more than one child, and they will have varied individual behavioural changes that this survey might not have accurately reflected. However, these limitations do not affect the main outcomes of the study, as this short and quick survey was useful as a general assessment tool, which can aid in situations where feedback is needed in a short period of time.

## Conclusions

The parents or guardians of school students were included in the current survey to study the effects of the pandemic on their children’s health-related behaviours. This study sheds light on the importance of monitoring populations across all demographics with a special focus on children when considering potential future pandemics. The study showed clear changes in the lifestyle and health-related behaviours of school students as indicated by their parents. The evidence in this study highlights the effects of quarantine and the importance of having alternative solutions and control measures. Further research is needed to investigate these changes and their effects to help design targeted innovative educational programs, especially during disaster situations such as pandemics and lockdowns, when the need to deliver education remotely becomes urgent. It is important to draw the attention of governments and health care authorities to the need for educating parents and children to adopt healthier habits at home, facilitating the transition between different pandemic response phases and developing programs to support the adoption of healthier habits. Furthermore, it is essential to address online learning issues such as weight gain, visual and back problems, and mental health problems. Online wellbeing and physical education lessons can be designed, and students and their parents must be educated on the importance of a healthy diet and appropriate sleep patterns. Education on posture and the importance of taking breaks from screens can be included as part of the health education curriculum in schools. Conducting more studies to properly investigate these interventions and determine their effects is recommended.

## Data Availability

The datasets generated and/or analysed during the current study are not publicly available due to federal privacy and data sharing policies but are available from the corresponding author upon reasonable request.

## Abbreviations

UAE: United Arab Emirates
MOHAP: Ministry of Health and Prevention
MOE: Ministry of Education
ADEK: Abu Dhabi Department of Education and Knowledge
ADPHC: Abu Dhabi Public Health Center
MVPA: Moderate to vigorous physical activity
